# Genomic profiles of primary and metastatic esophageal adenocarcinoma identified via digital sorting of pure cell populations: results from a case report

**DOI:** 10.1186/s12885-018-4789-4

**Published:** 2018-09-12

**Authors:** Federica Isidori, Deborah Malvi, Silvia Fittipaldi, Claudio Forcato, Isotta Bozzarelli, Claudia Sala, Giovanni Raulli, Antonia D’Errico, Michelangelo Fiorentino, Marco Seri, Kausilia K. Krishnadath, Elena Bonora, Sandro Mattioli, EAC-BAGH group, Sandro Mattioli, Maria Luisa Lugaresi, Elena Bonora, Federica Isidori, Isotta Bozzarelli, Antonietta D’Errico, Deborah Malvi, Gastone Castellani, Claudia Sala, Kausilia K. Krishnadath, Roberto Fiocca, Luca Mastracci, Jari Räsänen, Henna Söderström

**Affiliations:** 1 PhD program in Cardio-Nephro-Thoracic Sciences, University of Bologna, Bologna, Italy; 2 Unit of Medical Genetics, Department of Medical and Surgical Sciences (DIMEC), University of Bologna, Policlinico St. Orsola-Malpighi Hospital, Bologna, Italy; 3 Department of Experimental, Diagnostic and Specialty Medicine (DIMES), Institute of Oncology and Transplant Pathology, University of Bologna, Policlinico St. Orsola-Malpighi Hospital, Bologna, Italy; 4 Menarini Silicon Biosystems, Bologna, Italy; 5 Department of Physics and Astronomy, University of Bologna, Bologna, Italy; 6 Unit of Pathology – AUSL Romagna, Ravenna, Italy; 7 Center for Experimental and Molecular Medicine, Academic Medical Center, Department of Gastroenterology and Hepatology, Amsterdam, The Netherlands; 8 Division of Thoracic Surgery, Department of Medical and Surgical Sciences, University of Bologna and Maria Cecilia Hospital, Cotignola, Italy; 9 Department of Medical and Surgical Sciences (DIMEC) Alma Mater Studiorum, University of Bologna, Via G. Massarenti 9, 40138 Bologna, Italy

**Keywords:** Esophageal adenocarcinoma, Next generation sequencing, Digital cell sorting

## Abstract

**Background:**

We report on a female patient who underwent primary radical resection for a stage 2B Her-2-positive Barrett’s-type esophageal adenocarcinoma (EAC). Despite Her-2 targeted therapy, her disease recurred and required repeated metastectomies.

**Case presentation:**

Digital cell sorting and targeted sequencing of cancer sub-clones from EAC and metastases revealed a completely mutated *TP53*, whereas the sorted stromal cells were wild-type. Her-2 amplification was significantly lower in the metastases when the patient became therapy-resistant.

**Conclusions:**

The mechanism of therapy resistance illustrated by this case could only be detected through accurate analysis of tumor sub-populations.

Investigating tumor sub-populations of recurrent disease is important for adjusting therapy in recurrent EAC.

**Electronic supplementary material:**

The online version of this article (10.1186/s12885-018-4789-4) contains supplementary material, which is available to authorized users.

## Background

The incidence of esophageal adenocarcinomas (EACs) is increasing, and the survival rate is low, despite the adoption of aggressive therapeutic protocols [1]. EACs are characterized by a high mutational frequency and frequent somatic structural rearrangements (copy number variations, CNVs) [2, 3]. However, because of admixture of stromal cells the exact status of somatic cancer alterations is difficult to be determined. Next Generation Sequencing (NGS) technologies hold the potential to reveal the molecular underpinnings of tumor biology with the accuracy required for clinical implementation. However, when the input DNA is a mixture of normal and tumor there is an inherent trade-off between sensitivity and specificity, further complicated by the fact that the tumor DNA derive from subpopulations with different genetic characteristics. This can dilute the signal from the variant alleles/copy number alterations to values close to the background noise or below the detection limit. We exploited an automatic sorting system enabling isolation of pure tumor cells for unambiguous genetic analysis with targeted NGS assays, to investigate at the molecular level a primary EAC and two metachronous metastases [4].

## Case presentation

We studied the case of a woman who underwent primary radical resection of an EAC, adjuvant chemo-radiotherapy, targeted chemotherapy and two-stage resection of chest metastases (Additional file 1). We combined next generation sequencing (NGS) with a high-throughput cell sorting technique to separate stromal from cancer cells and identified diverse somatic mutations underlying the primary EAC and metastases.

**Fig. 1:**
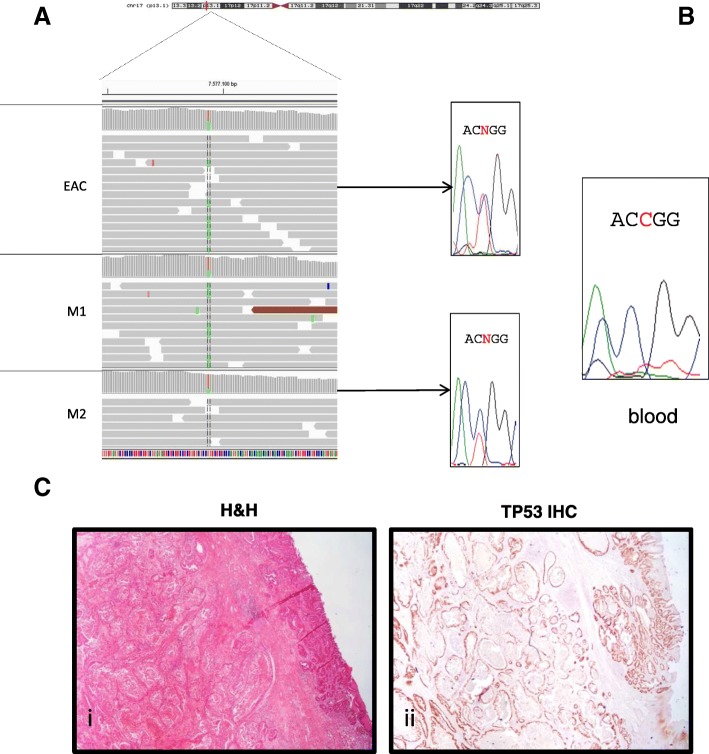
TP53 p.Arg282Trp mutation. **a** Representation of the *TP53* mutation (Integrative Genomic Viewer, IGV) and (**b**) Sanger sequencing of EAC, metastasis and blood. **c** (i) Histological appearance of the primary EAC; (ii) TP53-immunoreactivity (low power magnification)

**Fig. 2:**
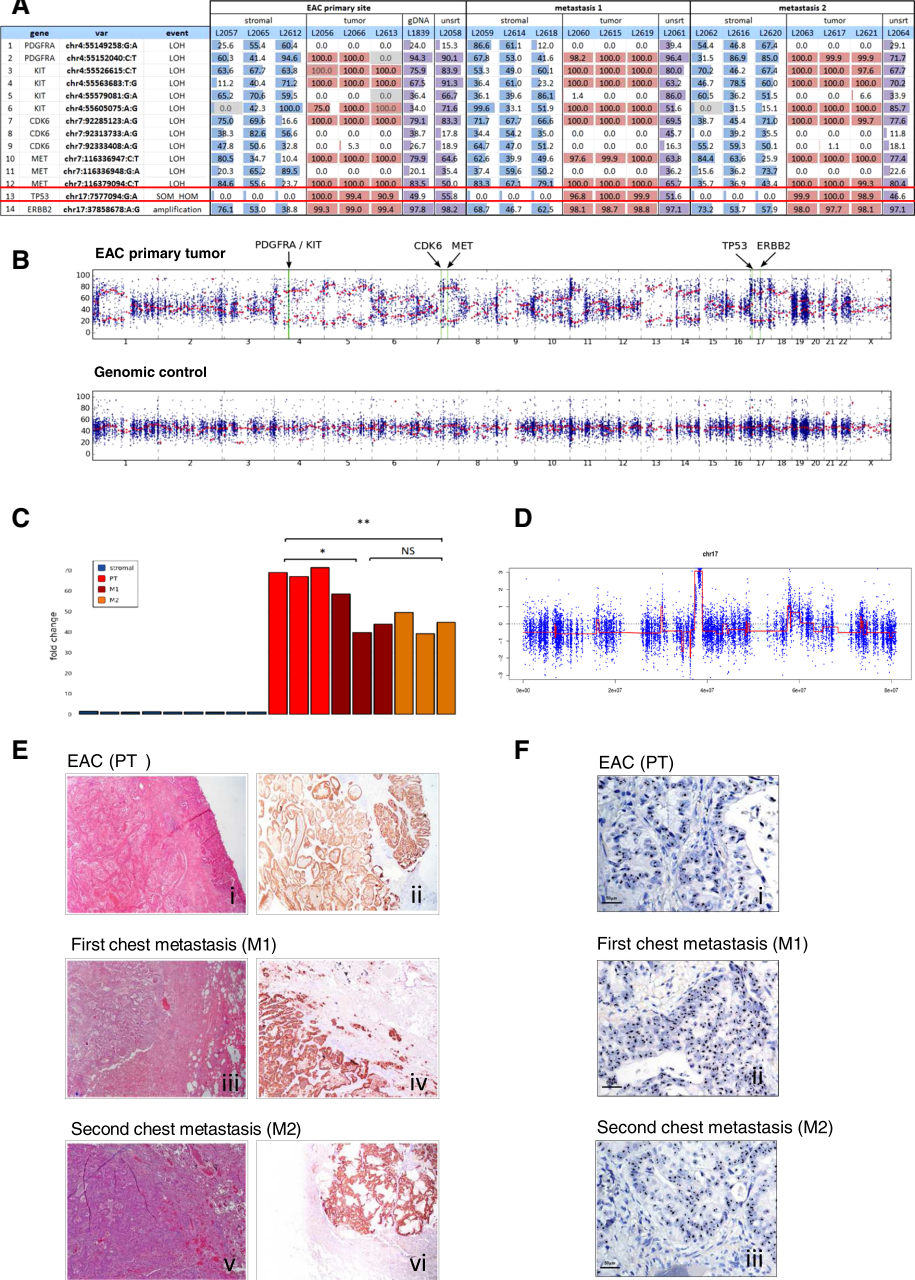
Variant identification in sorted cell populations (stromal and tumor) from primary EAC and chest metastases. **a** Relevant variants in the sorted pure populations of tumor (red), stromal (blue) cells and unsorted fractions (violet). Numeric values represent the alternative allele frequency. Table cells with gray background highlight positions with very low coverage. **b** BAF plot obtained using WES data of the primary EAC and a control female individual (WES performed on genomic DNA derived from peripheral blood). In the tumor track, the green lines highlight the positions of genes with putative LOH events detected using the OncoSeek panel. While the control profile shows a flat signal centered around 50%, as expected for a normal germline DNA, EAC tumor profile highlights several consistent regions with abnormal allele frequency, describing putative copy-number altered regions. Given the high variability of allele frequency, due to the relative low coverage in WES, a local smoothing on 20 Mb-long regions, represented by red dots, was calculated specifically to mitigate the frequency variability and to give a sharper idea of copy-number alterations at genome-level. **c** *Her-2* fold-change in all sorted pure populations (stromal and tumor). Histogram of CNV differences in the primary EAC and metastases. * = *p* < 0.05,** = *p* < 0.001, NS = not significant (ANOVA test). **d** *Her-2* Copy-number analysis using EAC WES data on unsorted material. **e** (i) Histological appearance and Her-2-immunoreactivity in the primary EAC (i-ii), M1 (iii-iv) and M2 (v-vi) metastases. **f** *Her2* cluster amplification detected by Ventana’s Her2 SISH test in primary EAC (i), M1 (ii) and M2 (iii) metastases. Clusters are represented by black areas in the nuclei (normal signal: black dots)

Whole Exome Sequencing (WES) of the primary EAC and the two metachronous chest metastases revealed a somatic heterozygous *TP53* missense mutation (chr17:g.7577094G > A,NP_000537.3: p.Arg282Trp, rs28934574, Fig. 1a), which was absent in the patient’s blood DNA (Fig. 1b). Cancer cells showed homogenous clusters in the primary tumor and metastases (Fig. 1ci). Intense immune-histochemical staining for p53, as detected in the EAC tumor area compared to no staining in the normal counterparts (Fig. 1cii), was consistent for a *TP53* missense mutations [5]. Using a selective sorting technology to separate cancer from stromal cell populations, we isolated 9 cancer and 9 stromal populations (Additional file 1: Table S1). Targeted NGS performed using the OncoSeek panel revealed that *TP53* was completely mutated in the EAC and metastatic clusters, while wild-type in the stromal cells (Fig. 2a – row 13). The purity of the sorted samples also detected several loss-of-heterozygosity (LOH) events involving the cancer-related genes on chromosome 4 *PDGFRA, KIT* (Fig. 2a – row 1–6), *CDK6* and *MET* on chromosome 7 in the primary EAC and metastases (Fig. 2a – row 7–12). Despite the lower purity of WES data, the B-allele frequency (BAF) profiles were consistent with the identified LOH events (Fig. 2b). Analysis of Copy Number Alteration (CNA), evaluated using the WES data on the whole primary tumor and metastases, showed a clear *Her-2* amplification, shared by primary tumor and both metastasis (Additional file 1: Table S2) and an additional gain of the chromosomal region 6q21–22.33 (18 Mb), that in the second chest metastasis generated a focal amplification (39 copies) spanning *RNF146* and *ECHDC1* genes (Additional file 2: Figure S1A-C, Additional file 1: Table S2). *ECHDC1* copy gains are present in COSMIC in two cases of esophageal cancer (COSG94494, COSMIC; http://cancer.sanger.ac.uk/).

SNP-based phylogenetic analysis with SNPhylo [6], using the WES data, revealed that the two chest metastases diverged in two branches derived from the primary lesion. Each metastasis presented the same phylogenetic distance from primary EAC, given by the sum of each branch length between the two samples (Additional file 2: Figure S1D).

The *Her-2* rs1565923A > G intronic variant showed a ~ 100% frequency in the tumor populations and unsorted samples, suggesting a high level of copy-gains (Fig. 2a – row 14). In concordance, CNV analysis of the sorted cell populations revealed also high level of *Her-2* amplification in the tumor subpopulations, confirmed by the WES data on unsorted material (Fig. 2b).

All sorted stromal cell populations did not carry this amplification (Fig. 2c, d). Interestingly, in the sorted cell populations the *Her2* fold-change decreased from ≅70-fold in the primary tumor to approximately ≅45-fold in the two recurrent chest metastases that subsequently developed. The fold-changes were not significantly different between the metastases (one-way ANOVA, *p* < 0.01 M2/PT, *p* < 0.05 M1/PT, Fig. 2c).

Using routine diagnostic techniques, no differences were observed in Her-2-immunostaining between the primary lesion and chest metastases (Fig. 2ei-vi). In situ hybridization (ISH) using a silver staining (SISH) method [7], showed clusters of *Her-2* amplification in the primary and the metastatic tumor sites, with a *Her-2/CEP17* ratio > 2 and *Her-2* copy number > 6 (clusters). Differences between the samples could not be appreciated with SISH, since all samples presented amplification clusters (Fig. 2fi-iii).

## Discussion

*TP53* mutations are considered early genetic events in Barrett’s esophagus associated with an increased risk of progression to cancer [2, 3, 8]. In general, it is difficult to assess whether a somatic mutation involves only one copy of a gene or both alleles in whole tumor tissue samples, due to stromal cell contamination. According to our data, the *TP53* mutant allele was completely mutated in the primary cancer, indicating that the *TP53* locus might have been involved in an early LOH event, which can explain the homozygous state of the p.Arg282Trp mutation. This mutation is frequently reported in several cancers (COSM10704, COSMIC; http://cancer.sanger.ac.uk/) and, as constitutive mutation, causes Li Fraumeni syndrome (ClinVar id12347; https://www.ncbi.nlm.nih.gov/clinvar/), an inherited cancer disease, characterized by autosomal dominant inheritance due to heterozygous mutations in *TP53*, with early onset and multiple tumors within an individual, including soft tissue sarcomas and osteosarcomas, breast cancer, brain tumors, leukemia and adrenocortical carcinoma (OMIM #151623). In our case, the variant was somatic, as indicated by its absence from peripheral blood-derived DNA (Fig. 1b).

The complete loss of TP53 wild-type protein in the studied tumor provides a significant impact on prognosis and therapeutic options, since the p.Arg282Trp mutation abolishes specific DNA binding, allowing evasion from apoptosis and accelerating tumor progression [8]. The pharmacological reactivation of mutant TP53 emerged as a promising strategy using molecules that restore its wild-type activity, such as APR-246/PRIMA-1Met, which is already under clinical trials for different cancers, including EAC [9, 10]. This molecule restores TP53 activity in presence of missense mutations and regulates several TP53-related pathways [9]. Therefore, precise identification of the *TP53* mutational status in EAC could be instrumental for selecting more efficient therapies. In the present case, the *TP53* mutation was shared by the EAC primary tumor and metastases (suggestive of an early origin) and we propose that restoring TP53 wild-type activity could be effective for metastases. From a technical perspective, the high-throughput sorting of the tumor cells led to the identification of somatic alterations without a “diluting” effect due to the presence of normal stromal cells. In addition, the capability of sorting pure stromal cells provides a convenient internal control [4]. Analyzing only the tumor but not matched normal tissue can yield many false-positive alterations that are not specific to the patient’s tumor. When matched normal tissue is unavailable, as may be the case for archival samples, this method can provide a valuable surrogate.

Standard tests measure *Her-2* CNV to guide the use of the anti-Her-2 drug trastuzumab in patients with metastatic disease secondary to gastric/gastroesophageal cancer, since ~ 24% of gastroesophageal adenocarcinomas overexpress *Her-2* [11]. However, trastuzumab-responsive patients may develop resistance, due to Her-2-dependent mechanisms such as the overexpression of proteins that mask the Her-2 receptor (e.g., MUC1), *Her-2* alternative splicing, or Her-2-independent mechanisms [12]. In the present case, compared to the primary tumor, the *Her-2* copy numbers detected by the selective cell sorting coupled to NGS were significantly lower in the metastases developed after trastuzumab therapy (Fig. 2a). This decrease was not detected by SISH, which showed clusters of the *Her-2* amplified region in the primary and metastatic tissues. Notably, the areas of metastatic tissues sampled for the CNV and ISH tests were morphologically homogeneous. The lower copy number in the metastases indicates a selection of sub-clones more resistant to treatment, although the histological appearance of the cells in the tumor areas was homogeneous. CNV analysis with WES data also showed a gain in *RNF146*-*ECHDC1* copy number, in the second metastasis. *RNF146* encodes for a E3 ubiquitin ligase ring finger protein 146, a critical regulator of Wnt/β-catenin signaling, whose overexpression, for example reported in non-small cell lung cancer, enhanced cell growth, invasion, and survival [13, 14]. *ECHDC1* encodes for a proofreading enzyme involved in lipid metabolism, with an increased expression observed in resistant bladder cancer cells [15]. We hypothesize that the acquired *RNF146*-*ECHDC1* copy gain in the cells giving raise to the second metastasis, coupled to the loss of cells with *HER2* amplification, might contributes to resistance and progression in metastatic EAC cancer. Sub-clone molecular heterogeneity is also revealed by the detection of somatic events in other cancer genes at different degrees.

## Conclusions

Digital cell sorting and omics-technologies in a Barrett’s-type EAC and two metachronous metastases revealed: (1) the true tumor cell mutational status of the somatic mutations and CNVs; (2) a progressive reduction of *Her-2* copy-gains in the two recurrent metastases compared to the primary tumor, not detectable by ISH.

We demonstrated that a genomic dissection of EAC and recurrent metastases could identify the tumor cell mutational status, as in this case for a *TP53* mutation and *Her2* copy-gains. Our pilot study took advantage of an available target panel for cancer-related genes, for the study of specific mutations in digitally-sorted cell populations from formalin-embedded tissue biopsies. Notably, a WES approach in unsorted material could identify additional CNVs in the different samples, but not the true tumor cell mutational status. It is therefore of key importance to apply whole exome/genome approaches to sorted cell populations from formalin-embedded tissue samples, in order to gain a global view of all the tumor alterations, as it is already applied for circulating tumor cells or fresh tumor tissues [16].

The incorporation of genomic differences in cancer cell sub-populations with currently available clinical variables can further stratify patients, in order to select the ones with highest risk of malignant progression for targeted therapies.

## Additional files

Additional file 1: Supplementary methods and metarials. (DOCX 79 kb)

Additional file 2: **Figure S1***.* Plots of Copy Number Analysis and phylogenetic tree using WES data. A 18 Mb region on chromosome 6 (q21-22.33) is indicated (red box), where CNV analysis identified a copy gain in PT (A) and M1 (B), as reported in Additional file 1: Table S2. (C) In the second chest metastasis (M2) a focal amplification was detected in the 6q22.33 region, spanning *RNF146* and *ECHDC1* genes (black arrowhead). (D) SNPhylo analysis results, showing the genetic distance between the three tumor samples. Numbers indicate the branch length from central node. The distance between two tumors is equal to the sum of their branch length. Analysis was performed according to [6]. (PDF 137 kb)

## Abbreviations

BAF: B-Allele Frequency
CNA: Copy Number Alteration
CNV: Copy Number Variation
EAC: Esophageal Adenenocarcinoma
ISH: In Situ Hybridization
LOH: Loss of Heterozigosity
NGS: Next Generation Sequencing
WES: Whole Exome Sequencing

## References

[1] Brown LM, Devesa SS, Chow WH (2008). Incidence of adenocarcinoma of esophagus among white Americans by sex, stage and age. J Natl Cancer Inst.

[2] Secrier M, Li X, De Silva N, Eldridge MD, Contino G, Bornschein J (2016). Mutational signatures in esophageal adenocarcinoma define etiologically distinct subgroups with therapeutic relevance. Nature.

[3] Cancer Genome Atlas Research Network (2017). Integrated genomic characterization of oesophageal carcinoma. Nature.

[4] Bolognesi C, Forcato C, Buson G, Fontana F, Mangano C, Doffini A (2016). Digital sorting of pure cell populations enables unambiguous genetic analysis of heterogeneous formalin-fixed paraffin embedded tumors by next generation sequencing. Sci Rep.

[5] Taylor NJ, Nikolaishvili-Feinberg N, Midkiff BR, Conway K, Millikan RC, Geradts J (2016). Rational manual and automated scoring thresholds for the immuhistochemical detection of TP53 missense mutations in human breast carcinomas. Immunohistochem Mol Morphol.

[6] Lee TH, Guo H, Wang X, Kim C, Paterson AH (2014). SNPhylo: a pipeline to construct a phylogenetic tree from huge SNP data. BMC Genomics.

[7] Dietal M, Ellis IO, Hofler H, Kreipe H, Moch H, Dankof A (2007). Comparison of automated silver enhanced in situ hybridisation (SISH) and fluorescence ISH (FISH) for the validation of HER2 gene status in breast carcinoma according to the guidelines of the American Society of Clinical Oncology and the College of American Pathologists. Virchows Arch.

[8] Contino G, Vaughan TL, Whiteman D, Fitzgerald RC (2017). The evolving genomic landscape of Barrett's esophagus and esophageal adenocarcinoma. Gastroenterology.

[9] Bykov VJN, Zhang Q, Zhang M, Ceder S, Abrahmsen L, Wiman KG (2016). Targeting of mutant p53 and the cellular redox balance by APR-246 as strategy for efficient cancer therapy. Front Oncol.

[10] APROC (ClinicalTrials.gov Identifier:NCT02999893).

[11] Samson P, Lockhart AC (2017). Biologic therapy in esophageal and gastric malignancies: current therapies and future directions. J Gastrointest Oncol.

[12] Rexer BN, Arteaga CL (2012). Intrinsic and acquired resistence to HER2-targeted therapies in HER2 gene-amplified breast cancer: mechanisms and clinical implications. Crit Rev Oncol.

[13] Zhu X, Xing R, Tan R, Dai R, Tao Q (2017). The RNF146 E3 ubiquitin ligase is required for the control of Wnt signaling and body pattern formation in Xenopus. Mech Dev.

[14] Gao Y, Song C, Hui L, Li CY, Wang J (2014). Overexpression of RNF146 in non-small cell lung cancer enhances proliferation and invasion of tumors through the Wnt/β-catenin signaling pathway. PLoS One.

[15] Asai S, Miura N, Sawada Y, Noda T, Kikugawa T, Tanji N (2018). Silencing of ECHDC1 inhibits growth of gemcitabine-resistant bladder cancer cells. Oncol Let.

[16] Ferrarini A, Forcato C, Buson G, Tononi P, Del Monaco V, Terracciano M (2018). A streamlined workflow for single-cells genome-wide copy-number profiling by low-pass sequencing of LM-PCR whole-genome amplification products. PLoS One.

