# Supplementary material for: Genomic profiles of primary and metastatic esophageal adenocarcinoma identified via digital sorting of pure cell populations: results from a case report
Source: BMC Cancer. 2018 Sep 12;18:889. doi: 10.1186/s12885-018-4789-4 (PMC6134594; doi:10.1186/s12885-018-4789-4)
Supplement: Supplementary file 1 — Supplementary methods and metarials. (DOCX 79 kb) [file 12885_2018_4789_MOESM1_ESM.docx]

**Supplementary Information**

**Supplementary methods**

**Whole-exome sequencing (WES)**

DNA extraction was performed with QIAMP DNA Mini Kit (Qiagen) starting from 3 sections 10-μm thick, obtained from FFPE (formalin fixed, paraffin embedded) blocks of primary tumor and chest metastases.

Genomic DNA was extracted from patient’s blood using the QIAGEN DNA Mini kit. Before proceeding to whole-exome sequencing, a quality control of retrieved DNA was performed with KAPA QC kit. 300 ng genomic DNA from each tissue sample was tagmented and enriched according to Nextera^®^ Rapid Capture Enrichment protocol with the coding exome exon kit (Nextera) and quantified libraries were sequenced on Illumina HiScan SQ platform at 100bp paired ends. Reads were checked with FastQC and aligned with BWA (bio-bwa.sourceforge.net) to the reference. Variant pathogenicity was evaluated with PolyPhen-2 (genetics.bwh.harvard.edu/pph2), Provean (provean.jcvi.org), MutationTaster (www.mutationtaster.org) for missense mutations, SplicePredictor, Human Splicing Finder, ESEfinder v3.0 (www.phenosystem.com) for putative splicing alterations.

Copy-number analyses were performed on WES data with EXCAVATOR2 software (sourceforge.net/projects/excavator2tool). In this case, in addition to the tested tumor sample, whole exome data derived from a normal sample was used as control.

**SNPhylo: phylogenetic analysis of primary tumor and metastases**

WES data were used for a phylogenetic analysis of the primary lesion and chest metastases. After converting the data in SNP File Format, we computed the phylogenetic tree with SNPhylo, using the default parameters, according to [6]. To calculate the phylogenetic distance, the program sums each branch length between the two samples.

**DEPArray™ sorting protocol**

Each section was collected in a nylon biopsy bag inside a 50 ml conical tube, dewaxed by three sequential 10 min incubations in xylene and then rehydrated via decreasing ethanol washes. After a wash in deionized water, section was immersed in 10 mM sodium citrate buffer (pH 6.4) for 5 min at room temperature and heat-treated in the same pre-warmed buffer for 1 h at 80°C. After cooling down at room temperature, the section was washed by three sequential 5 min incubations with RPMI medium (Life Technologies). In order to obtain a cell suspension, the section was incubated in 10 ml of 0.1% collagenase I-A (Sigma-Aldrich) and 0.1% dispase (Life Technologies) solution, at 37°C. The dissociation process was stopped after 45 min by placing the sample tube on ice. The cell suspension was resuspended by pipetting and transferred through a 30 μm mesh nylon filter into a 15 ml conical tube. Cell suspension was washed two times in ice-cold PBATw (PBS-1%BSA-0.05%Tween20 buffer) by centrifugation at 1,000 g for 5 min.

The cell pellet was resuspended in 1 ml of ice-cold PBATw and an aliquot of 5 × 105 cells was incubated with 100 μl of primary monoclonal antibody mixture containing anti-keratin MNF116, IgG1 (DAKO) (final concentration 3.2 μg/ml), anti-keratin AE1/AE3, IgG1 (Millipore–Chemicon) (final concentration 10 μg/ml) and anti-vimentin 3B4, IgG2a (DAKO, Glostrup, Denmark) (final concentration = 3.1 μg/ml) in PBATw. After 30 min at 4°C, cells were washed twice with ice-cold PBATw and 100 μl premixed secondary reagents Alexa Fluor® 488 Goat Anti-Mouse IgG1 (Life Technologies), final concentration = 2.5 μg/ml for keratin detection and Alexa Fluor® 647 Goat Anti-Mouse IgG2a (Life Technologies), final concentration = 2.5 μg/ml for vimentin detection, in PBATw was added to the pellet. Incubation for 60 min in the dark at 4°C was followed by washing two times with ice-cold PBATw. DNA staining solution containing 10 μM DAPI (Sigma-Aldrich) in PBATw was added and after an incubation of 30 min at 37°C cells were washed twice with PBATw by a 5 min centrifugation at 1,000 g and pellet was resuspended in the same buffer.

For DEPArray™ cell sorting, a small amount of the labelled cell suspension was washed twice with 1 ml of SB115 buffer (Menarini Silicon Biosystems). Pellet was resuspended in the same buffer and an aliquot, corresponding to about 24,000 cells, loaded into DEPArray™ A300K cartridge (Silicon Biosystems).

After identification and selection of cell populations showing the desired fluorescence patterns, a precise number of homogeneous cells from tumor and stromal populations, together with pools of mixed cells, were recovered in different PCR tubes. After lysis, reagents were added in the same tubes for preparing DEPArray™ OncoSeek libraries. Details of cell recoveries are summarized in Supplementary Table 1.

**DEPArray™ OncoSeek genetic analysis**

Each DEPArray™ OncoSeek library was diluted 1:10.000 and then quantified in triplicate by the qPCR using the KAPA Library Quantification kit (KAPA Biosystems), following the user’s manual instruction. Finally, the quantification was adjusted for the average library size of 243 bp. All the libraries were pooled and NGS was carried out using MiSeq v2 (150 PE) reagents on MiSeq instrument (Illumina), according to the manufacture’s protocol.

FASTQ paired-end reads were trimmed using Cutadapt (cutadapt.readthedocs.io) according to the manufacturer’s protocol to remove synthetic primers on overlapping amplicons. Trimmed reads were aligned on human reference genome (hg19) using BWA software (bio-bwa.sourceforge.net). Alignment and coverage statistics were obtained using Samtools (samtools.sourceforge.net) and BEDTools (bedtools.readthedocs.io/en/latest) packages. After a filtering step for discarding partial or poor alignments and unmapped reads, variant calls were obtained using LoFreq software (csb5.github.io/lofreq). Resulting variants were annotated using Ensembl Variant Effect Predictor (www.ensembl.org/info/docs/tools/vep/index.html).
Copy-number alteration analysis on OncoSeek data was performed using sorted populations as tests and a set of stromal cell pools from different samples as controls. For copy-number calling, reads mapping on target amplicons of DEPArray™ OncoSeek panel were counted. Then, read counts were normalized using a 2-step procedure: 1) between-samples normalization, using total number of aligned reads, and 2) within-sample normalization, using a LOWESS fitting of read counts respect to the first component explaining >90% variation between regions in control samples. Fold changes were computed dividing normalized counts in test samples by the baseline, represented by the median value of normalized counts per amplicon across control samples. Final copy-number calls per gene were obtained calculating the median of fold changes of all gene-specific amplicons.

Statistical significance of fold-change difference among primary tumor, first and second chest metastases replicates was determined using ANOVA test (one-way analysis of variance), with a significance level of 5%.

**LOH detection from WES data on genomic DNA**

Exome data from the DNA-extracted tumor sample were obtained through enrichment with Nextera capture kit and sequencing with Illumina HiScan SQ, as mentioned above. LoFreq software was used to get variant calls, which were later annotated with Ensembl VEP. Variant frequencies and annotations were used to produce a B-allele frequency plot for investigating the presence of LOH regions. Given the lack of the matching normal sample and the subsequent inability to figure out germline heterozygous variants, only highly polymorphic sites (GMAF > 0.1) were used in the plot. Moreover, variant frequencies greater than 90% or lower than 10% were filtered out to exclude germline homozygous SNPs and background noise. Removal of these variants does not affect the LOH identification, since the expected tumor frequencies of LOH variants in unsorted samples are lower than 100% and greater than 0% due to contamination of normal tissue. In addition, to improve the interpretation of frequency trends along the genome, allele frequencies were smoothed using a kernel density estimation (KDE) in 20Mb overlapping bins. LOH regions were visually predicted focusing on stretches of consecutive variants with frequencies deviating from heterozygous state (~50%).

**Immunohistochemistry**

From the FFPE blocks, immunohistochemistry was performed automatically with Benchmark XT^®^ immunostainer (Ventana). Antibodies for the following antigens will be applied: p53, HER2.

The immunohistochemical analysis has been validated through positive controls (as an external positive control put on the slide) and negative control (by omitting the primary antibody).

The immunoreactivity for the HER2 protein has been scored applying the HER2 gastric cancer score.^25^ The expression of p53 protein was defined as hyper-expressed if there was evidence of strong and diffuse nuclear immunoreactivity.

**In Situ Hybridization (ISH)**

In Situ Hybridization was performed using the dual-color silver/chromogenic *in situ* hybridization test for *Her-2* gene and chromosome 17 (centromeric probe) copy number variations was performed by INFORM HER2 Dual ISH DNA Probe Cocktail on a Benckmark Ultra instrument (Ventana Medical Systems/Roche Diagnostics, Mannheim, Germany). Forty non-overlapping nuclei were counted at ×600 magnification.

**Table S1**: **Summary statistics regarding DEPArray™ recoveries and OncoSeek sequencing.**

DNA index is a measure of DNA content, determined comparing the integral-intensity DAPI of tumor population with that of stromal fraction, used as reference. All tumor populations show a hyperdiploid profile, compared to the expected diploid stromal profile. EAT (Effective Addressable Template) is a number estimating the usable template for DEPArray™ OncoSeek assay and can be considered a measure of sample quality. It is determined according to the following formula: n.cells x DNA_index x 2 x FFPE_QC_score.


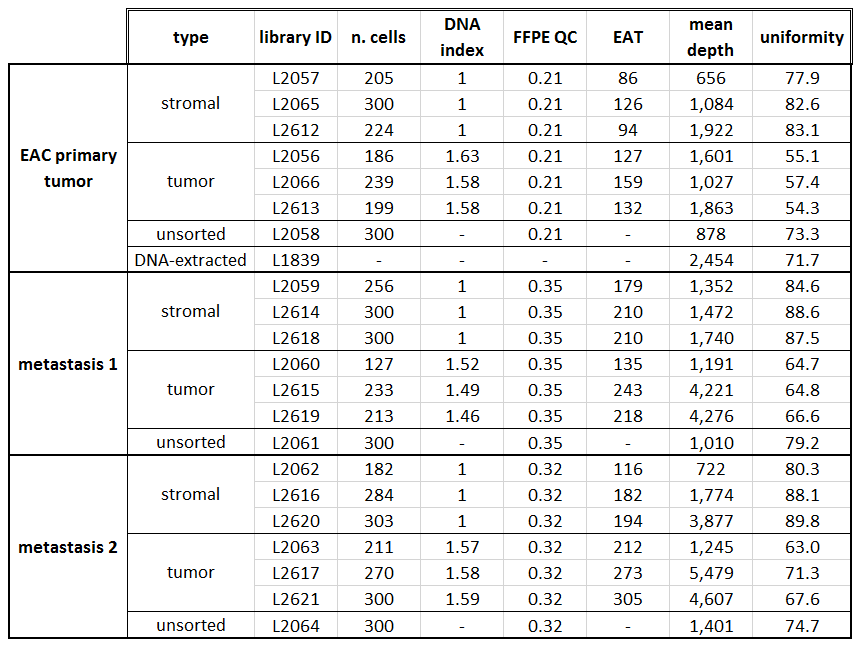


**Table S2: EXCAVATOR2 output for CNV on chromosome 6 and 17.** EXCAVATOR2 results reports chromosome [CHROM], start [START], end [END] (positions compared to the reference genomic sequence hg19), segment length of a specific copy number [SEGMENT], copy number value (rounded to nearest integer) [CN], call probability inferred by FastCall algorithm [ProbCall], for EAC primary tumor (PT), first chest metastasis (M1) and second chest metastasis (M2).

|  | CHROM | START | END | SEGMENT | CN | ProbCall |
| --- | --- | --- | --- | --- | --- | --- |
| EAC (PT) | 6 | 109294558 | 109761795 | 467 kb | 4 | 0.933 |
|  | 6 | 123573555 | 123905087 | 331 kb | 3 | 0.895 |
|  | 17 | 37263657 | 38948823 | 1,7 Mb | 17 | 0.999 |
| M1 | 6 | 109308744 | 109761795 | 453 kb | 4 | 0.945 |
|  | 6 | 123573555 | 123905087 | 331 kb | 4 | 0.780 |
|  | 6 | 123905288 | 123905288 | 15 Mb | 3 | 0.560 |
|  | 17 | 37295904 | 38948823 | 1,7 Mb | 11 | 0.988 |
| M2 | 6 | 108843497 | 109761795 | 918 kb | 3 | 0.933 |
|  | 6 | 123545236 | 123868517 | 323 kb | 3 | 0.891 |
|  | 6 | 127601482 | 127636156 | 34 kb | 39 | 0.999 |
|  | 17 | 37263657 | 38948823 | 1,7 Mb | 11 | 0.999 |
