# Supplementary figures and images for: Genomic profiles of primary and metastatic esophageal adenocarcinoma identified via digital sorting of pure cell populations: results from a case report
Source: BMC Cancer. 2018 Sep 12;18:889. doi: 10.1186/s12885-018-4789-4 (PMC6134594; doi:10.1186/s12885-018-4789-4)

Supplementary Figure 1

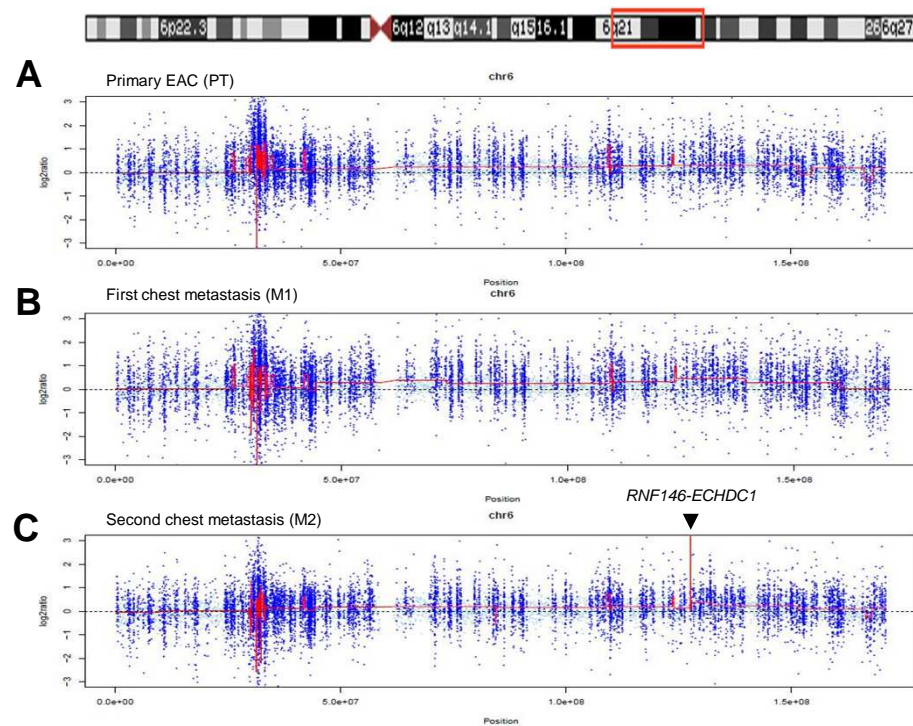

**D**

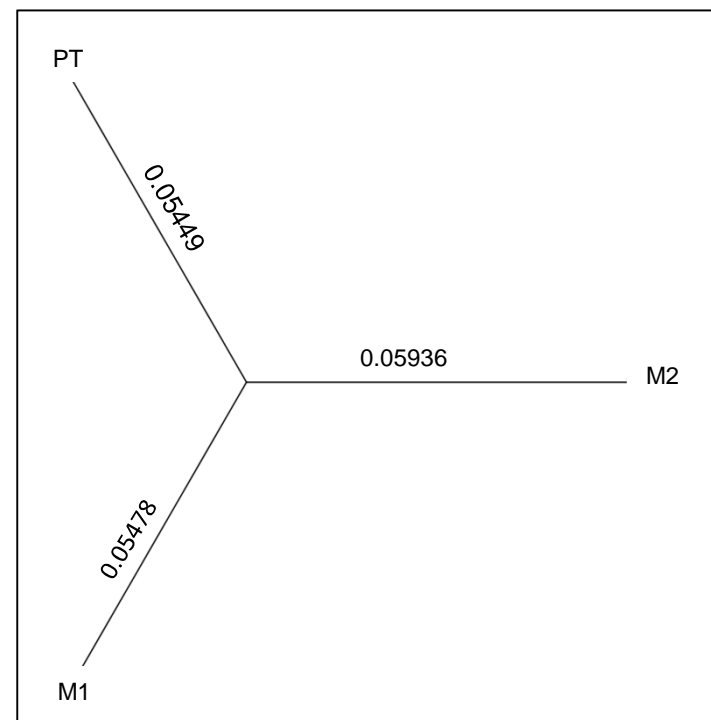

Supplement: Supplementary file 2 — Figure S1. Plots of Copy Number Analysis and phylogenetic tree using WES data. A 18 Mb region on chromosome 6 (q21-22.33) is indicated (red box), where CNV analysis identified a copy gain in PT (A) and M1 (B), as reported in Additional file 1: Table S2. (C) In the second chest metastasis (M2) a focal amplification was detected in the 6q22.33 region, spanning RNF146 and ECHDC1 genes (black arrowhead). (D) SNPhylo analysis results, showing the genetic distance between the three tumor samples. Numbers indicate the branch length from central node. The distance between two tumors is equal to the sum of their branch length. Analysis was performed according to [6]. (PDF 137 kb) [file 12885_2018_4789_MOESM2_ESM.pdf]
